# Impact of compliance with infection management guidelines on outcome in patients with severe sepsis: a prospective observational multi-center study

**DOI:** 10.1186/cc13755

**Published:** 2014-03-03

**Authors:** Frank Bloos, Daniel Thomas-Rüddel, Hendrik Rüddel, Christoph Engel, Daniel Schwarzkopf, John C Marshall, Stephan Harbarth, Philipp Simon, Reimer Riessen, Didier Keh, Karin Dey, Manfred Weiß, Susanne Toussaint, Dirk Schädler, Andreas Weyland, Maximillian Ragaller, Konrad Schwarzkopf, Jürgen Eiche, Gerhard Kuhnle, Heike Hoyer, Christiane Hartog, Udo Kaisers, Konrad Reinhart

**Affiliations:** 1 Department of Anesthesiology and Intensive Care Medicine, Jena University Hospital Jena, 07740, Germany; 2 The Integrated Research and Treatment Center for Sepsis Control and Care (CSCC), Jena University Hospital Jena, 07740, Germany; 3 Institute for Medical Informatics, Statistics and Epidemiology, University of Leipzig Leipzig, Germany; 4 Department of Surgery and the Li Ka Shing Knowledge Institute, St Michael’s Hospital, University of Toronto, Toronto, Ontario, Canada; 5 Infection Control Program, Geneva University Hospitals and Medical School, Geneva, Switzerland; 6 Department of Anesthesiology and Intensive Care Medicine, University Hospital Leipzig, Leipzig, Germany; 7 Department of Internal Medicine, University Hospital Tübingen, Tübingen, Germany; 8 Department of Anesthesiology and Intensive Care Medicine, Charité Berlin, Berlin, Germany; 9 Department of Anesthesiology and Intensive Care Medicine, Bundeswehrkrankenhaus Berlin, Berlin, Germany; 10 Department of Anesthesiology, University Hospital Ulm, Ulm, Germany; 11 Department of Anesthesiology, Intensive Care Medicine, and Pain Therapy, Vivantes Klinikum Neukölln, Berlin, Germany; 12 Department of Anesthesiology and Intensive Care Medicine, University Medical Center Schleswig-Holstein, Campus Kiel, Kiel, Germany; 13 Department of Anesthesiology, Intensive Care Medicine, Emergency Medicine, and Pain Therapy, Hospital Oldenburg, Oldenburg, Germany; 14 Department of Anesthesiology and Intensive Care Medicine, University Hospital Carl Gustav Carus, Dresden, Germany; 15 Department of Anesthesiology and Intensive Care Medicine, Hospital Saarbrücken, Saarbrücken, Germany; 16 Department of Anesthesiology and Intensive Care Medicine, St. Georg Hospital Eisenach, Eisenach, Germany; 17 Department of Anesthesiology and Intensive Care Medicine, SRH Waldklinikum Gera, Gera, Germany; 18 Institute of Medical Statistics, Computer Sciences and Documentation, Jena University Hospital, Jena, Germany

## Abstract

**Introduction:**

Current sepsis guidelines recommend antimicrobial treatment (AT) within one hour after onset of sepsis-related organ dysfunction (OD) and surgical source control within 12 hours. The objective of this study was to explore the association between initial infection management according to sepsis treatment recommendations and patient outcome.

**Methods:**

In a prospective observational multi-center cohort study in 44 German ICUs, we studied 1,011 patients with severe sepsis or septic shock regarding times to AT, source control, and adequacy of AT. Primary outcome was 28-day mortality.

**Results:**

Median time to AT was 2.1 (IQR 0.8 – 6.0) hours and 3 hours (-0.1 – 13.7) to surgical source control. Only 370 (36.6%) patients received AT within one hour after OD in compliance with recommendation. Among 422 patients receiving surgical or interventional source control, those who received source control later than 6 hours after onset of OD had a significantly higher 28-day mortality than patients with earlier source control (42.9% versus 26.7%, *P* <0.001). Time to AT was significantly longer in ICU and hospital non-survivors; no linear relationship was found between time to AT and 28-day mortality. Regardless of timing, 28-day mortality rate was lower in patients with adequate than non-adequate AT (30.3% versus 40.9%, *P* < 0.001).

**Conclusions:**

A delay in source control beyond 6 hours may have a major impact on patient mortality. Adequate AT is associated with improved patient outcome but compliance with guideline recommendation requires improvement. There was only indirect evidence about the impact of timing of AT on sepsis mortality.

## Introduction

In the treatment of severe sepsis, timely and effective antimicrobial therapy (AT) as well as source control is crucial and has become a key element in the resuscitation bundles proposed by the Surviving Sepsis Campaign (SSC) [1]. The SSC guidelines recommend obtaining blood cultures and applying intravenous broad-spectrum antimicrobials within 1 hour after the onset of severe sepsis or septic shock; the guidelines also recommend initiating surgical source control within 12 hours [1]. Numerous studies have shown that a delay of AT and inappropriate initial AT in this condition is associated with poor outcome [2-6]. One retrospective study in patients with septic shock suggests an increase of patient mortality between 7 and 8% per hour within the first 6 hours after onset of arterial hypotension [3]. There is also evidence that delayed surgery is associated with lower survival rates [7-9], but the appropriate time frame remains poorly defined [10].

In numerous retrospective and before-and-after studies, improved adherence to the sepsis bundles was associated with improved patient outcome [4,11-14]. A meta-analysis suggests that among the individual elements of the resuscitation bundle, timely and appropriately administered antimicrobials are the most important predictors for survival [15]. However, compliance with sepsis guideline recommendations is poor [16]. In a Spanish multicenter trial, only 18.4% of the studied patients received AT within the first hour of severe sepsis or septic shock. The administration of antimicrobials within 1 hour was independently associated with a lower risk of hospital death [6].

Application of antimicrobials without microbiological evidence of infection might be associated with an unfavorable outcome [17]. Likewise, starting AT in patients with severe sepsis before obtaining blood cultures was among the factors that were associated with higher hospital mortality [4]. Compliance with the recommendation to draw two pairs of blood cultures before AT, however, was only in the range of 54.4 to 64.5% of patients [4,13,18].

Most of the previous studies investigating the timing of antimicrobials and the compliance with the SSC sepsis bundles reported only ICU mortality rates, focused mainly on patients treated in the emergency department or in the ICU, and did not evaluate the impact of delayed source control on patient outcome [3,5,17,19,20]. However, many of these patients may be admitted also from general wards or the operating room and may require effective surgical and other measures of source control. We therefore extended our assessment of infection control measures to include these patients as well. The primary aim of our cohort study was to prospectively test the hypothesis that a delay in AT and source control after onset of sepsis-related organ dysfunction impacts patient outcome. In addition, we aimed to assess compliance with recent best-practice recommendations for the diagnosis and therapy of sepsis.

## Methods

### Study design

This prospective study was designed as a longitudinal multicenter observational cohort study in 42 German hospitals to determine the time to AT, surgical source control and compliance with sepsis recommendations related to AT in patients with suspected severe sepsis or septic shock and its impact on 28-day, ICU, and hospital mortality. Participation of hospitals was voluntary but was restricted to hospitals involved in the primary care of sepsis patients and committed to participate in a quality improvement process. Hospitals without ICUs were excluded from this study. Potential study centers were recruited by regional and national research and quality improvement networks. The objectives of the study, inclusion and exclusion criteria, and the documentation procedures were discussed in several national meetings before the start of the study. The study was designed as a pragmatic study with a minimal case report form to allow for the participation of hospitals without research staff. This study served as a run-in study for a cluster-randomized trial assessing whether a multifaceted educational program accelerates the onset of AT and improves survival (Medical Education for Sepsis Source Control and Antibiotics MEDUSA, ClinicalTrials.gov Identifier NCT01187134).

### Patients

Between December 2010 and April 2011, all consecutive adult patients treated in the ICU for proven or suspected infection with at least one new organ dysfunction related to the infection were eligible for inclusion. Organ dysfunctions were defined as follows: acute encephalopathy, thrombocytopenia defined as a platelet count <100,000/μl or a drop in platelet count >30% within 24 hours, arterial oxygen partial pressure <10 kPa (75 mmHg) when breathing room air or partial pressure of arterial oxygen/fraction of inspired oxygen ratio <33 kPa (<250 mmHg), renal dysfunction defined as oliguria (diuresis ≤0.5 ml/kg body weight/hour) despite adequate fluid resuscitation or an increase of serum creatinine more than twice the local reference value, metabolic acidosis with a base excess < −5 mmol/l or a serum lactate >1.5 times the local reference value, and arterial hypotension defined as systolic arterial blood pressure <90 mmHg or mean arterial blood pressure <70 mmHg for >1 hour despite adequate fluid loading or vasopressor therapy at any dosage to maintain higher blood pressures [21]. Patients who received initial infection control measures for sepsis in another hospital and patients who did not receive full life-sustaining treatment were excluded. The study was reviewed and approved by the local ethics committees, which waived the need for informed consent because of the observational nature of the study (see Acknowledgements). The study was also approved by the local data protection boards.

### Data collection

Onset of severe sepsis or septic shock was defined as the time of first infection-related organ dysfunction as documented in the patient file. Patient location at time of onset of severe sepsis was defined as the patient location where the first infection-related organ dysfunction was documented. For patients who developed severe sepsis outside the ICU, this could be the prehospital setting, the emergency department, the hospital ward, or the operating room. Time and type of first AT as well as pre-existing AT were also recorded from the medical records. Any AT prescribed up to 24 hours before the onset of organ dysfunction but for the current infectious episode was considered previous AT. Perioperative antimicrobial prophylaxis was not regarded as specific AT for sepsis. Change of empirical AT was assessed on day 5. Initial AT was defined as inadequate if escalation had occurred within the first 5 days. For each patient, a blinded arbitrator assessed whether the initial AT complied with German guideline recommendations [22]. Source control was defined as removal of an anatomic source of infection either by surgery or intervention (that is, computed tomography-guided drainage). Source control was defined as inadequate if the technical procedure was unsuccessful. Time to source control was obtained from the medical record. Other factors included serum lactate and procalcitonin at the time of onset of severe sepsis, number of blood culture sets taken, and ICU and hospital mortality. Severity of disease was assessed by the Simplified Acute Physiology Score II and the Sequential Organ Failure Assessment score on the day of sepsis diagnosis [23,24].

Data were collected by a web-based electronic case report form using OpenClinica® (OpenClinica, LLC, Waltham, MA, USA). Data integrity was confirmed by data checks within the database, resulting in queries to the investigator where applicable. Additionally, onset of infection-related organ dysfunction and subsequent AT were checked for plausibility by the MEDUSA study staff and discussed with the study center staff where applicable.

### Statistical analysis

The primary endpoint was survival status at day 28 after onset of severe sepsis. Categorical data are expressed as absolute or relative frequencies; the chi-square or Fisher's exact test was used for inferential statistics. Continuous data are expressed as the median and interquartile range; the Mann–Whitney U test was used for inferential statistics. Missing data were not replaced by calculation. We divided patients according to the timing of antimicrobial treatment into the following groups: previous AT, 0 to 1 hours, 1 to 3 hours, 3 to 6 hours and >6 hours [6]. Patients were grouped by time to source control into two groups: within 6 hours or >6 hours [25]. Odds ratios (OR) with 95% confidence intervals (CI) for the risk of death within 28 days depending on time to AT or time to source control were calculated by univariate and multivariate logistic regression only in those patients were treatment was started after onset of organ dysfunction. In patients with sepsis, prior research has identified the initial Sequential Organ Failure Assessment score, age, and serum lactate [20] as confounders for the risk of death. These parameters were therefore included in the multivariable logistic regression analysis to calculate adjusted ORs. In addition, indication for source control, escalation as well as de-escalation of empirical AT within 5 days, presence of community acquired infection, focus of infection, and state of blood culture withdrawal were only included into the final model if they were associated with 28-day mortality at *P* < 0.20. Goodness of fit was assessed by the *c*-statistic and the Hosmer-Lemeshow test.

## Results

### Participating centers and patients

Hospital and ICU characteristics of the 44 participating centers are shown in Table 1. A total of 1,048 patients were included during the 5-month study period. Of these, 37 patients were excluded for missing values in 28-day mortality or time to AT, resulting in 1,011 evaluable patients. Approximately one-half of the patients (*n* = 504, 49.9%) were treated for surgical reasons. Organ dysfunctions on the day of study inclusion were shock (*n* = 632, 76.4%), lactacidosis (*n* = 424, 51.3%), acute renal failure (*n* = 307, 37.2%) thrombocytopenia (*n* = 238, 28.8%), pulmonary dysfunction (*n* = 492, 65.8%), and septic encephalopathy (*n* = 338, 41.2%). A total 85.1% of the patients had more than one organ failure. Patient characteristics are shown in Table 2. The overall 28-day mortality was 34.8%; ICU mortality and hospital mortality were 33.0% and 41.4%, respectively. There was no association of academic versus nonacademic hospitals or hospital size with 28-day mortality.

**Table 1:** **Characteristics of participating hospitals and ICUs (*n*** **= 44)**

**Hospital**	**Data**
University hospital	10 (22.7%)
Level of care	
Primary care hospitals	14 (31.8%)
Secondary care hospitals	12 (27.3%)
Tertiary care hospitals	18 (40.9%)
Hospital beds	581 (416 to 1,055)
Number of ICUs	2 (1 to 4)
Inhospital biochemistry laboratory	43 (97.7%)
Inhospital microbiology department	30 (68 to 2%)
ICU beds	15 (10 to 29)
ICU patients per annum	1,200 (900 to 2,050)
Emergency department	
Emergency department available	37 (84.1%)
Lactate available within 1 hour	38 (86.4%)
Broad-spectrum antibiotics available	36 (81.8%)
Prehospital emergency physician available	39 (88.6%)

Data are shown as number (percentage) for categorical data or median (interquartile range) for continuous data.

**Table 2:** Patient characteristics

	**28-day survivors (*n*** **= 659)**	**28 day nonsurvivors (*n*** **= 352)**	**All patients (*n*** **= 1,011)**	***P *** **value**
Male	413 (62.7%)	221 (62.8%)	634 (62.7%)	0.972
Age (years)	68 (55 to 75)	72 (64 to 79)	69 (58 to 77)	<0.001
ICU admission				0.254
Elective surgery	74 (11.2%)	42 (11.9%)	116 (11.5%)	
Emergency surgery	255 (38.7%)	118 (33.5%)	373 (36.9%)	
Trauma	10 (1.5%)	5 (1.4%)	15 (1.5%)	
Medical	304 (46.1%)	183 (52%)	487 (48.2%)	
Other	16 (2.4%)	4 (1.1%)	20 (2%)	
Patient location at onset of sepsis				0.426
Emergency department	93 (14.1%)	47 (13.4%)	140 (13.9%)	
ICU	366 (55.6%)	214 (60.8%)	580 (57.4%)	
Operating theatre	63 (9.6%)	22 (6.2%)	85 (8.4%)	
Hospital ward	80 (12.2%)	41 (11.6%)	121 (12%)	
Prehospital	12 (1.8%)	4 (1.1%)	16 (1.6%)	
IMC	44 (6.7%)	24 (6.8%)	68 (6.7%)	
Infection				0.22
Community acquired	300 (45.5%)	143 (40.6%)	443 (43.8%)	
ICU/IMC acquired	170 (25.8%)	91 (25.9%)	261 (25.8%)	
Hospital acquired	189 (28.7%)	118 (33.5%)	307 (30.4%)	
Source of infection (multiple responses possible)				
Intra-abdominal	228 (34.7%)	138 (39.2%)	366 (36.3%)	0.167
Pneumonia	221 (33.7%)	130 (36.9%)	351 (34.9%)	0.311
Urogenital	92 (14.0%)	30 (8.5%)	122 (12.1%)	0.011
Upper airway	59 (9.0%)	24 (6.8%)	83 (8.2%)	0.228
Bones/soft tissue	47 (7.2%)	25 (7.1%)	72 (7.1%)	0.966
Other	67 (11.7%)	38 (10.7%)	105 (10.4%)	0.863
Unknown	25 (3.8%)	25 (7.1%)	50 (5%)	0.022
ICU length of stay (days)	11 (4 to 26)	7 (4 to 13)	11 (4 to 24)	0.003
Hospital length of stay (days)	32 (19 to 52)	18 (14.5 to 21.5)	32 (19 to 52)	0.011
SOFA score	9 (7 to 11)	11 (9 to 15)	10 (8 to 12)	<0.001
SAPS II	45 (34 to 56)	54 (45 to 68)	48 (37 to 60)	<0.001
Lactate maximum (mmol/l)	2.3 (1.3 to 3.9)	4.0 (2 to 8.1)	2.7 (1.5 to 4.9)	<0.001
Procalcitonin (ng/ml)	6.3 (1.6 to 25)	7.0 (2.2 to 21.9)	6.5 (1.8 to 22.8)	0.465

Categorical data are expressed as absolute or relative frequencies (number (percentage); continuous data are expressed as median (interquartile range). SOFA score, lactate, and procalcitonin refer to the day of sepsis onset. *P* value refers to differences between survivors and nonsurvivors. IMC, intermediate care unit; SAPS, Simplified Acute Physiology Score; SOFA, Sequential Organ Failure Assessment.

### Timing and adequacy of antimicrobial therapy

**Figure 1:**
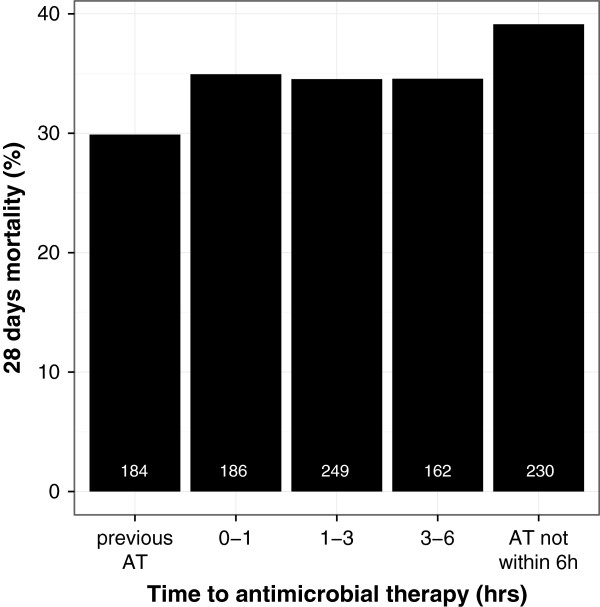
**Twenty-eight-day mortality according to time to antimicrobial therapy.** Numbers in the bars represent number of patients in this group. Previous AT, patients who received antimicrobial therapy (AT) before onset of infection-related organ dysfunction.

Three hundred and seventy (36.6%) patients received antimicrobials within 1 hour of the onset of organ dysfunction; among these, 186 (18.4%) patients received antimicrobials in the first hour and 184 patients (18.2%) received antimicrobials prior to onset of organ dysfunction. Six hundred and forty-one (63.3%) patients received their first AT more than 1 hour after onset of organ dysfunction (Figure 1). Median time to AT was 2.1 (interquartile range: 0.8 to 6) hours in all patients and 2.8 (interquartile range: 1.0 to 2.8) hours for patients that received their AT after development of organ dysfunction. Median times by location where sepsis developed were 2.8 (1 to 7.2) hours in intermediate care wards, 2.7 (1.5 to 5. 2) hours in hospital wards, 2.3 (0.6 to 7.3) hours in ICUs, 2.2 (1.4 to 3.0) hours prehospital, 2.0 (1.0 to 3.5) hours in emergency departments, and 1.1 (0.2 to 4.7) hours in operating theatres. Three hundred and twenty-one patients were admitted to the ICU with septic shock. Median time to AT was 2.1 (1.0 to 5.0) hours in this subgroup. The 28-day mortality was 34.9% in the 186 patients who received AT during the first hour and 36.2% in 641 patients when AT was given more than 1 hour after onset of the first sepsis-related organ dysfunction (*P* = 0.76). For the subgroup of 370 patients who received AT within 1 hour, 28-day mortality was 32.4% compared with 36.2% for those 641 patients receiving delayed AT (*P* = 0.227). There was no linear relationship between time to AT and 28-day mortality (Figure 1). There was also no association between time to AT and risk of death within 28 days (OR per hour increase of time to AT: 1.0 (95% CI: 1.0 to 1.0), *P* = 0.482) in those 849 patients that received their AT after the first organ dysfunction. Considering a mean time to AT of 6.1 (± standard deviation 9.5) hours, the data would allow one to identify an effect OR of 1.02 per hour increase with an alpha level of 0.05 and a power of 0.8.

The 28-day mortality was lower (29.9%) in patients who developed severe sepsis or septic shock while being treated with antimicrobials compared with a mortality rate of 35.9% in the patients who were treated only after diagnosis, although the difference was not statistically significant (*P* = 0.121). Time to first AT was longer for nonsurvivors than survivors (Table 3). This difference reached statistical significance for ICU mortality (*P* = 0.023) and hospital mortality (*P* = 0.02) but not for 28-day mortality (*P* = 0.112). For 763 (75.5%) patients, the initial empirical therapy complied with the German recommendations for AT. The 28-day mortality was 34.9% in the compliant group and 34.3% in the noncompliant group (*P* = 0.869).

**Table 3:** Time to antimicrobial therapy and source control according to survival

	**Survivors**	**Nonsurvivors**	***P *** **value**
Time to antimicrobial therapy (hours)			
28-day survival	2.0 (0.6 to 5.6)	2.5 (1.0 to 6.6)	0.112
	(*n* = 659)	(*n* = 352)	
ICU survival	2.0 (0.7 to 5.4)	2.8 (0.9 to 7.0)	0.023
	(*n* = 667)	(*n* = 329)	
Hospital survival	2.0 (0.6 to 5.1)	2.8 (0.9 to 7.0)	0.020
	(*n* = 581)	(*n* = 329)	
Time to source control (hours)			
28-day survival	2.0 (−0.5 to 10.1)	5.7 (0.4 to 18.0)	0.004
	(*n* = 286)	(*n* = 139)	
ICU survival	2.0 (−0.6 to 9.1)	6.0 (0.5 to 19.9)	<0.001
	(*n* = 286)	(*n* = 132)	
Hospital survival	2.0 (−0.5 to 9.3)	5.5 (0.4 to 18.9)	0.001
	(*n* = 249)	(*n* = 166)	

Data are shown as median and interquartile range.

The most frequently used empirical antimicrobial agents were piperacillin/tazobactam or ampicillin/sulbactam (26.7%), followed by imipenem/cilastatin (8.2%) and meropenem (7.8%). Empirical AT was escalated within the first 5 days in 423 (41.9%) patients. Therapy was escalated more often in patients receiving antimicrobials not in compliance with guideline recommendations (125 of 245, 51%) than in patients treated in compliance with guidelines (298 of 762, 39.1%; *P* < 0.01). For 96 patients (9.5%), AT was de-escalated within 5 days. The 28-day mortality rate was 22.9% in patients with de-escalation compared with 36.0% in patients without de-escalation (*P* = 0.01).

For 587 (58.1%) patients, AT was deemed adequate. In patients with inadequate AT, the 28-day mortality rate was significantly higher compared with adequately treated patients (40.9% vs. 30.3%, *P* < 0.001; Table 4). This increased risk was also evident when patients received AT within the first hour: in patients receiving AT within 1 hour, 28-day mortality was 26.9% (32/119) when this AT was deemed adequate compared with 48.5% (32/66) for patients with inadequate AT; likewise, in patients receiving AT later than 1 hour, 28-day mortality was 31.2% (146/468) for adequate compared with 42.7% (118/278) for an inadequate AT (*P* < 0.001). Among 588 patients not requiring surgical or interventional source control, 101/335 (30.1%) patients with adequate AT and 112/253 (44.3%) patients with inadequate AT died within 28 days (*P* < 0.001).

**Table 4:** Patient population stratified by adequacy of empirical antimicrobial therapy

	**Adequate AT (*n*** **= 587)**	**Inadequate AT (*n*** **= 423)**	***P *** **value**
Age	70 (58 to 77)	69 (57 to 76.2)	0.458
Infection			0.048
Community acquired	276 (47%)	166 (39.2%)	
ICU/IMC acquired	144 (24.5%)	117 (27.7%)	
Hospital acquired	167 (28.4%)	140 (33.1%)	
28-day mortality	178 (30.3%)	173 (40.9%)	<0.001
SOFA score	9 (7 to 12)	10 (8 to 12)	0.002
ICU length of stay	8.5 (4 to 22)	14 (5 to 28)	<0.001
Lactate maximum (mmol/l)	2.5 (1.5 to 4.7)	2.9 (1.6 to 5)	0.09
Procalcitonin (ng/ml)	6.3 (1.7 to 21)	6.6 (1.9 to 27.9)	0.497

Inadequacy was defined as escalation of AT within 5 days. Categorical data are expressed as absolute and relative frequencies (number (percentage)) and are compared by the Pearson test; continuous data are expressed as median (interquartile range) and are compared by the Kruskal–Wallis test. SOFA score, lactate, and procalcitonin refer to the day of sepsis onset. AT, antimicrobial therapy; IMC, intermediate care ward; SOFA, Sequential Organ Failure Assessment.

By multivariable analysis, inadequate empirical AT (OR (95% CI): 1.44 (1.05 to 1.99)) as well as age (OR (95% CI): 1.04 (1.03 to 1.06)), initial Sequential Organ Failure Assessment score (OR (95% CI): 1.18 (1.13 to 1.24)) and maximum serum lactate levels on the day of diagnosis of severe sepsis or septic shock (OR (95% CI): 1.09 (1.05 to 1.14)) were significantly associated with an increased risk of death. Adjusted for these and further covariates, a delay in the administration of AT more than 1 hour after onset of organ dysfunction (OR (95% CI): 0.96 (0.69 to 1.33)) was not associated with an increased 28-day mortality (Table 5). Likewise, no association between time to AT and outcome was seen in the subgroup of patients without need for surgical or interventional source control (Table 5).

**Table 5:** Multivariate logistic regression model for the impact of patient-related factors on 28-day mortality

**Variable**	**Odds ratios (95% CI)**	***P *** **value**
**All patients (*n*** **= 725)**^**a**^		
Time to antimicrobial therapy >1 hour^b^	0.81 (0.54 to 1.23)	0.323
Initial SOFA score^c^	1.19 (1.13 to 1.26)	<0.001
Age^d^	1.04 (1.03 o 1.06)	<0.001
Maximum lactate (day 1)^e^	1.09 (1.04 to 1.14)	<0.001
Intra-abdominal focus	1.08 (0.75 to 1.57)	0.670
Urogenital focus	0.65 (0.36 to 1.14)	0.143
Unknown focus	1.26 (0.57 to 2.78)	0.574
Community-acquired infection	0.89 (0.65 to 1.22)	0.484
Inadequate empiric antimicrobial therapy	1.44 (1.05 to 1.99)	0.026
No de-escalation of antimicrobials within 5 days	1.17 (0.66 to 2.14)	0.597
**Surgical source control required (*n*** **= 234)**^**f**^		
Time to antimicrobial therapy >1 hour^b^	0.80 (0.38 to 1.72)	0.552
Initial SOFA score^c^	1.19 (1.08 to 1.31)	<0.001
Age^d^	1.06 (1.03 to 1.08)	<0.001
Maximum lactate (day 1)^e^	1.08 (1.00 to 1.13)	0.046
Time to source control >6 hours	2.36 (1.22 to 4.71)	0.012
Intra-abdominal focus	1.08 (0.54 to 2.18)	0.822
Urogenital focus	0.43 (0.12 to 1.34)	0.165
Unknown focus^g^	–	–
Community-acquired infection	1.08 (0.58 to 2.04)	0.800
Inadequate empiric antimicrobial therapy	1.17 (0.61 to 2.24)	0.646
No de-escalation of antimicrobials within 5 days	0.94 (0.33 to 2.81)	0.909
**No surgical source control required (*n*** **= 424)**^**h**^		
Time to antimicrobial therapy >1 hour^b^	0.69 (0.39 to 1.21)	0.189
Initial SOFA score^c^	1.19 (1.11 to 1.28)	<0.001
Age^d^	1.04 (1.02 to 1.06)	<0.001
Maximum lactate (day 1)^e^	1.12 (1.05 to 1.20)	0.001
Intra-abdominal focus	1.72 (0.93 to 3.19)	0.083
Urogenital focus	0.95 (0.47 to 1.86)	0.875
Unknown focus	1.67 (0.70 to 3.98)	0.243
Community-acquired infection	1.03 (0.64 to 1.65)	0.904
Inadequate empiric antimicrobial therapy	1.52 (0.95 to 2.42)	0.078
No de-escalation of antimicrobials within 5 days	2.71 (1.02 to 8.40)	0.061

Adjusted odds ratios and 95% confidence intervals (CIs) for 28-day mortality. Only patients with initiation of antimicrobial therapy as well as source control after development of organ dysfunction and with complete observations in all variables are entered into this analysis. Parameters not included due to result in the monovariate analysis: surgical or interventional source control required (*P* = 0.223), status of blood culture withdrawal (*P* = 0.779), pulmonary focus (*P* = 0.491), other focus than intra-abdominal, pulmonary, urogenital or unknown (*P* = 0.691). Inadequate antimicrobial therapy was defined as escalation of empiric antimicrobial therapy within 5 days. All models showed a good separation of the outcome (*c*-statistic >0.7) and a good calibration (*P* > 0.05 in the Hosmer–Lemeshow test (pHLT)). SOFA, Sequential Organ Failure Assessment. ^a^Goodness of fit: *c* = 0.76, pHLT = 0.904. ^b^Against previous antimicrobial therapy and antimicrobials within 1 hour after infection-related onset of organ dysfunction. ^c^Per point increase. ^d^Per year. ^e^Per mmol/l. ^f^Goodness of fit: *c* = 0.79, pHLT = 0.733. ^g^Insufficient sample size in this subgroup. ^h^Goodness of fit: *c* = 0.77, pHLT = 0.887.

### Blood culture testing

Blood cultures were taken before AT in 649 (64.2%) patients, and 48.8% of these cultures were positive. In 269 patients (41.4% of patients from whom blood cultures were drawn), only one set of blood cultures was obtained. In the 317 positive blood cultures, 187 (62.1%) showed Gram-positive bacteria, 127 (42.2%) showed Gram-negative bacteria, and 20 (6.6%) showed fungi; 32 (10.6%) of the positive blood cultures revealed more than one pathogen.

Among the 317 patients with a positive blood culture, 63.3% received antimicrobials before onset of organ dysfunction and 103 (33.7%) received antimicrobials after onset of organ dysfunction. The 28-day mortality in these groups was 35.9% and 31.5%, respectively (*P* = 0.440).

### Time and adequacy to source control

Surgical (84.8%) or interventional (15.9%) source control was performed in 422 patients: overall median time to source control was 3 (–0.1 to 13.7) hours and 6 (2 to 20) hours, respectively, in those patients where source control was initiated after development of organ dysfunction (*n* = 314). One hundred and fifty-eight of 314 patients (50.3%) received source control within 6 hours after onset of infection-related organ dysfunction. The time to source control was significantly longer in nonsurvivors than in survivors (Table 3). In 55 patients (13.3%), source control was assessed as being inadequate. The 28-day mortality was 65.5% in patients with inadequate source control compared with 26.7% in patients with adequate source control (*P* <0.01). There was no direct relationship between time to source control and risk of death within 28 days (OR per hour increase of time to source control: 1.0 (95% CI: 1. to 1.0), *P* = 0.725). Patients who had surgical source control delayed for more than 6 hours had a significantly higher 28-day mortality (42.9% vs. 26.7%, *P* <0.001); this delay was independently associated with an increased risk of death (Table 5). There was neither a statistically significant interaction nor a collinearity between time to AT and time to source control.

## Discussion

This prospective observational trial included 1,011 evaluable patients with severe sepsis from a large group of academic and nonacademic hospitals. The main finding of this study was that surgical source control within the first 6 hours was associated with 16% lower 28-day mortality. This finding is of interest since the SSC guidelines recently increased the window for source control from 6 hours [25] to 12 hours [1] after diagnosis. This decision is based on a single study in patients with necrotizing soft tissue infection, where a delay of surgery >14 hours was associated with an increased risk of death [7]. However, this study by Boyer and colleagues did not examine the effect of shorter delays on mortality. While current data suggest that delayed surgery adversely affects outcome [26,27], studies to allow the determination of an optimal time point of surgical source control are rare. A retrospective analysis of patients with fecal peritonitis did not confirm a relationship between duration until source control and mortality [28]. However, overall mortality in this study was very low with 19.1% and only 24-hour time intervals were reported. Our data are more consistent with an observational study of children reporting that all patients who received surgical debridement for necrotizing fasciitis at later than 3 hours died [8]. Likewise, a study in patients with perforated peptic ulcers found that each hour delay in surgical source control increases 30-day mortality by 2% [9]. Clearly, more data on the relationship between time to source control and patient outcome are needed. In the interim, surgical source control should be performed as soon as possible.

Our observation that early AT of the underlying infection of sepsis before onset of organ dysfunction is associated with a trend towards lower 28-day mortality in the range of 6% supports the importance of early recognition and antimicrobial treatment of infection underlying sepsis [1]. The finding that the median time to antimicrobial treatment was about 40 minutes shorter in survivors than in nonsurvivors confirms other studies [18,29]. Median times to antimicrobial administration were 2.1 hours after diagnosis of severe sepsis or septic shock and thus exceeded guideline recommendations [1]. Similar delays have been reported in other studies [5,13,18,20,30,31]. In contrast to our data, a number of studies demonstrated an association between patient outcome and time to AT in patients with severe infections [2,32-35]. Like other studies [6,20], we could not confirm the data of Kumar and coworkers in patients with septic shock that suggested an increase of 7.6% in hospital mortality per hour delay in AT [3]. This may be related to differences in the patient population or study methodology. Kumar and colleagues focused their work on patients with septic shock and observed a median time to AT of 6 hours – three times longer than what we and other studies have observed [3].

There are some other considerations that may explain the different findings about time to AT and its association with mortality. Firstly, some studies used the time until adequate AT [3,5,34] rather than time to first AT, as we did. We rather applied an approach similar to Puskarich and colleagues because it seems unreasonable to assess the quality of primary care with microbiological data that are not available for the treating physician at that time [20]; other studies also used this design [2,17,36]. Furthermore, the underlying pathogen may remain unknown and alternative definitions of adequacy such as guideline adherence [3,34] need to replace the microbiological definition of adequacy anyhow. Secondly, the definition of the starting time for the duration until AT is defined significantly different across the available studies and includes hospital [33,35] or ICU admission [2], onset of arterial hypotension [3,34], and the time when cultures were obtained [17,36]. We have chosen onset of infection-related organ dysfunction since this is a clinical feature that should trigger initiation of primary sepsis care. All of the chosen starting times may overlook that significant organ dysfunction occurred before the defined time. These considerations suggest that the investigation of the impact of timing of AT on patient outcome is limited in observational studies.

The concept of early empirical AT has recently been challenged. Puskarich and colleagues did not find an increase in mortality with each hour delay in AT in emergency department patients with septic shock [20]. In a before-and-after-study in critically ill surgical patients, AT initiated only after microbiological confirmation was associated with a lower mortality rate than early empirical AT [17]. However, the overall long delays to antimicrobial administration in both groups (11 and 17.7 hours, respectively) limit interpretation of results from that study [37].

In general, compliance with sepsis guideline recommendations was poor. Only one-third of patients received their first antimicrobial agent according to current guideline recommendations before or within 1 hour of diagnosis of severe sepsis. Blood cultures before AT were taken in 649 (64.2%) patients; however, two sets of blood cultures were obtained in only one-half of these patients. Choice of antimicrobials complied with German recommendations for empirical AT [22] in 75% of cases. Nevertheless, in about 40% of patients the treating physicians considered first AT as inadequate and escalated AT within the first 5 days. Overall, 28-day mortality of these patients was considerably increased. The association between adequacy of AT and patient outcome remained significant regardless of whether AT was given earlier or later than 1 hour after onset of severe sepsis. This was also true for the 588 patients not requiring surgical or interventional source control. Therefore it seems unlikely that AT was deemed inadequate and changed because the patient deteriorated for reasons unrelated to the microbiological inappropriateness of AT, such as inadequate surgical source control. Increased mortality in patients with inappropriate initial AT has also been observed in other studies [38,39]. Recent data from the EUROBACT study concluded that infections with multiresistant organisms are associated with a delay of appropriate AT and increased mortality [40].

Current guidelines recommend at least two sets of blood cultures before starting AT [1]. In our study, two-thirds of patients had blood cultures drawn before AT. However, only one set was drawn in about 50% of those patients. Drawing blood cultures before initiation of broad-spectrum antimicrobials was associated with a lower risk of death in the SSC database [4] but not in our study. This may be explained by the much larger sample size in the SSC database.

Our study has strengths and weaknesses. Strengths include the prospective data collection and multicenter design. Unlike previous studies, our study used short-term prospective data collection and is therefore not influenced by secular trends. Furthermore, reporting of times to AT not only in the ICU but also in other locations as well as outside the hospital and inclusion of medical centers with all levels of care increases the generalizability of our results. Although we enrolled over 1,000 patients, the sample size may not have been large enough to detect small differences in outcome; moreover, we cannot rule out that eligible patients were not included in the study because of limited resources. We also did not include patients who were not referred to the ICU. However, it is unlikely that many such patients were missed since in Germany the majority of patients with organ dysfunction are referred to an ICU or intermediate care unit.

We did not assess adequacy of AT by means of microbiological susceptibility testing results because many of the included hospitals lacked the staff to report such data for a study. Instead, we used the pragmatic approach to ask physicians to record any change of AT within 5 days, which was defined *a priori* as an indication of inadequate initial therapy. Despite the fact that we found this association also in nonsurgical patients, we cannot rule out that AT was changed because the patient deteriorated for reasons that were not related to the microbiological inappropriateness of AT. Except for serum lactate measurements, which were obtained in 95.2% of the patients at baseline, we did not assess the compliance with other guideline recommendations and therefore cannot rule out that mortality rates were potentially influenced by unmeasured effects; for instance, timely fluid resuscitation or appropriate use of other supportive measures.

## Conclusions

More data on the relationship between time to source control and patient outcome are needed. In the interim, surgical source control should be performed as soon as possible. Adequacy of empirical AT is important for the survival in sepsis, and choice of initial AT is an important decision in the therapy of these patients. There was only indirect evidence about the impact of timing of AT on sepsis mortality but evidence about this issue varies significantly among the available studies. Randomized controlled trials are thus necessary to further elucidate the impact of AT timing on survival. Quality improvement initiatives should not be restricted to severe sepsis but should also focus on the timely recognition and adequate treatment of infections to prevent their progress to severe sepsis.

## Key messages

•A delay of surgical or interventional source control of more than 6 hours was associated with increased mortality.

•Although survivors had a shorter time to AT than nonsurvivors, there was no significant association or linear relationship between time to AT and survival.

•An inadequate empiric AT was associated with an increased mortality.

•Compliance with guidelines regarding anti-infectious measures regarding timing and choice of empiric AT, withdrawal of blood cultures, and de-escalation of AT should be improved.

## Abbreviations

AT: antimicrobial therapy; CI: confidence interval; OR: odds ratio; SSC: Surviving Sepsis Campaign.

## Competing interests

The authors declare that they have no competing interests.

## Authors’ contribution

All authors made substantive intellectual contributions to the manuscript. FB and KR conceived and designed the study, drafted the manuscript, and were responsible for the grant funding. DaS, DT-R, HR, PS, RR, DK, KD, MW, ST, DiS, AW, MR, KS, JE, GK, and UK participated in the acquisition of the data, were responsible for the conduct of the study and helped to revise the manuscript. CE and HH participated in the study design and the statistical data analysis and helped to revise the manuscript. JCM, SH, and CH participated in the assessment of the data analysis and revised the manuscript. All authors read and approved the final manuscript.
